# Effects of gut microbiota–derived extracellular vesicles on obesity and diabetes and their potential modulation through diet

**DOI:** 10.1007/s13105-021-00837-6

**Published:** 2021-09-02

**Authors:** Ester Díez-Sainz, Fermín I. Milagro, José I. Riezu-Boj, Silvia Lorente-Cebrián

**Affiliations:** 1 Department of Nutrition, Food Science and Physiology/Center for Nutrition Research, Faculty of Pharmacy and Nutrition, University of Navarra, Pamplona, Spain; 2 Navarra Institute for Health Research (IdiSNA), Pamplona, Spain; 3 Centro de Investigación Biomédica en Red Fisiopatología de La Obesidad Y Nutrición (CIBERobn), Instituto de Salud Carlos III, Madrid, Spain; 4 Department of Pharmacology, Physiology and Legal and Forensic Medicine, Faculty of Health and Sport Science, University of Zaragoza, Zaragoza, Spain; 5 Instituto Agroalimentario de Aragón-IA2 (Universidad de Zaragoza-CITA), Zaragoza, Spain; 6 Aragón Health Research Institute (IIS-Aragon), Zaragoza, Spain

**Keywords:** Exosomes, Probiotics, microRNA, Dysbiosis, Inflammation, *Akkermansia muciniphila*

## Abstract

Obesity and diabetes incidence rates are increasing dramatically, reaching pandemic proportions. Therefore, there is an urgent need to unravel the mechanisms underlying their pathophysiology. Of particular interest is the close interconnection between gut microbiota dysbiosis and obesity and diabetes progression. Hence, microbiota manipulation through diet has been postulated as a promising therapeutic target. In this regard, secretion of gut microbiota–derived extracellular vesicles is gaining special attention, standing out as key factors that could mediate gut microbiota-host communication. Extracellular vesicles (EVs) derived from gut microbiota and probiotic bacteria allow to encapsulate a wide range of bioactive molecules (such as/or including proteins and nucleic acids) that could travel short and long distances to modulate important biological functions with the overall impact on the host health. EV-derived from specific bacteria induce differential physiological responses. For example, a high-fat diet–induced increase of the proteobacterium *Pseudomonas panacis*–derived EV is closely associated with the progression of metabolic dysfunction in mice. In contrast, *Akkermansia muciniphila* EV are linked with the alleviation of high-fat diet–induced obesity and diabetes in mice. Here, we review the newest pieces of evidence concerning the potential role of gut microbiota and probiotic-derived EV on obesity and diabetes onset, progression, and management, through the modulation of inflammation, metabolism, and gut permeability. In addition, we discuss the role of certain dietary patterns on gut microbiota–derived EV profile and the clinical implication that dietary habits could have on metabolic diseases progression through the shaping of gut microbiota–derived EV.

## Introduction

Gut microbiota is the set of microbes that colonize the gastrointestinal tract of mammals, establishing a mutualistic relationship with the host [115]. Gut microbiota is involved in a wide range of host essential processes, such as digestion and metabolism (i.e., fat storage, indigestible polysaccharide degradation, and therapeutic drug metabolization), development and physiology of the immune system, gut function (i.e., gut permeability regulation and maintenance of gut barrier integrity), angiogenesis, pathogen resistance, bone homeostasis, or behavior [77, 115]. It should be noted that several environmental factors could shape gut microbiota composition, including lifestyle issues, antibiotics, or diet, the latter being considered an important environmental factor that could influence gut microbiota composition [32, 115]. The alteration of gut microbiota homeostasis, commonly known as dysbiosis, has been associated with numerous diseases, such as colorectal cancer, inflammatory bowel disease, cardiovascular diseases, neurological disorders, autoimmune diseases, obesity, and diabetes [44, 115].

In particular, obesity prevalence is increasing dramatically and it has been estimated that about 57.8% of the world adult population will be overweight or obese by 2030 [29, 66]. Likewise, the global incidence rate of type 2 diabetes, which accounts for 90–95% of all cases of diabetes, is also rising: it has been predicted that it will increase from 6,059 cases *per* 100,000 in 2017 to 7,079 individuals *per* 100,000 in 2030 [39, 67]. The association between weight gain and type 2 diabetes risk is well known, by which the term “diabesity” has been coined to those cases when type 2 diabetes is caused by obesity [97, 108]. Obesity and type 2 diabetes are associated with an increased risk to develop a plethora of diseases, such as cardiovascular diseases [22, 54], mental disorders, [10, 12], or cancer [50], and they have also been linked with the severity of infectious diseases, such as tuberculosis or COVID-19 [60, 65].

Recent studies have proposed that gut microbiota dysbiosis could be involved in the onset and progression of obesity and diabetes [32, 112]. Thus, gut microbiota manipulation interventions are postulated as new promising approaches to treat these metabolic disorders, which include dietary interventions (such as administration of prebiotics, probiotics, symbiotics, or low-fat diets), fecal microbiome transplantation, or bariatric surgery [1, 32, 37, 94, 112, 129]. Nevertheless, a better understanding of the mechanisms underlying the interaction between gut microbiota and the host is mandatory in order to ultimately identify targets through which the gut microbiota could be effectively manipulated to potentially treat obesity and diabetes.

In recent years, extracellular vesicles (EVs) have emerged as crucial players in inter-kingdom communication, including those established between gut microbiota and mammals [11, 130]. EVs are cell-secreted particles identified in all domains of life (eukaryote, bacteria, and even archaea) that have the potential ability to regulate cell function in an autocrine or paracrine manner [130, 133]. In mammals, depending on their biogenesis and size, EVs have been classified mainly in exosomes, microvesicles, and apoptotic bodies [43]. Exosomes are EVs of endocytic origin that range in size from 30 to 150 nm [43]. By contrast, microvesicles have a diameter ranging from 100 to 1000 nm and are formed from plasma membrane; and apoptotic bodies range from 50 to 5000 nm and are originated from cells undergoing apoptosis [43]. Plants can also secrete EVs, whose biogenesis, content, and morphology are similar to mammalian exosomes, by which they are usually termed as “exosome-like nanovesicles” [33, 131]. EVs have been also identified in both Gram-negative and Gram-positive bacteria [80, 114]. Attending to their biogenesis, they have been termed as outer-outer-inner and explosive-outer membrane vesicles in Gram-negative bacteria, and cytoplasmic membrane vesicles in Gram-positive bacteria [122]. As summarized by Cuesta et al. [31], bacterial EVs share many similarities with eukaryotic EVs, including their size (10–400 nm), morphology (spherical particles), and physico-chemical properties (such as stability upon freezing and at 37 °C). However, several differences have been found regarding characteristics such as structure, tolerance to high temperatures, composition, and their biogenesis [31]. Of note, a common characteristic between eukaryotic and bacterial EVs is the ability to transport genetic material, including microRNAs (miRNAs) or miRNA-like molecules, which have been found in a wide range of EVs, such as mammalian exosomes, microvesicles and apoptotic bodies [103], plant exosome-like nanovesicles [132], and bacterial EVs [3].

EV cargo is configured by a wide range of bioactive molecules, including proteins, lipids, polysaccharides, RNA, and DNA [130, 133]. Among the different types of cargo encapsulated in EVs, miRNAs have acquired special notoriety, since it has been reported that they can modulate gene expression in unrelated organisms, including plants-animals, plants-bacteria, and animals-bacteria [78]. Remarkably, true miRNA-like molecules have been identified in bacteria [78]. These molecules are of similar size to eukaryotic miRNAs and can be encapsulated and secreted in EVs, which protect miRNAs from degradation [78]. In particular, gut microbiota-derived EVs have been associated with several host functions, including immune system regulation and cancer suppression and it has also been suggested that these EVs might have a role in gut-brain axis modulation, since it was reported that they could reach the central nervous system and regulate cerebral function [56, 70, 88]. In addition to displaying beneficial effects and their contribution to host homeostasis, the alteration of gut microbiota-derived EV profile has been associated with the progression of several diseases, such as HIV, inflammatory bowel disease, or cancer therapy–induced intestinal mucositis [123]. Furthermore, gut microbiota–derived EVs, in particular those secreted by *Paenalcaligenes hominis*, could even induce cognitive disorders like Alzheimer’s disease [81].

In this review, we will discuss the potential role of gut microbiota and probiotic-derived EVs on obesity, diabetes, and inflammation, and we will summarize the current pieces of evidence concerning the influence of diet on gut microbiota and probiotic-derived EVs and their potential implications of this interaction on host health.

## Role of gut microbiota and probiotic-derived extracellular vesicles on inflammation, obesity, and diabetes

Gut microbiota plays a crucial role in the modulation of host physiological processes whose alterations have been strongly linked to obesity and diabetes onset and progression (i.e., food intake, inflammation, metabolism, and intestinal barrier function) [20, 30, 53, 93]. Growing evidence suggests that gut microbiota–derived EVs could be relevant mediators in microbe-host cross-kingdom communication, playing part in gut microbiota-mediated intestinal homeostasis and ultimately being involved in the pathogenesis of metabolic diseases.

### Effects of gut microbiota and probiotic-derived extracellular vesicles on gut barrier fortification and inflammation suppression

Alvarez et al. [5] evidenced that EVs secreted by the probiotic strains of *Escherichia coli* Nissle 1917 (EcN) and ECOR63 could strengthen intact intestinal epithelial barrier by upregulating zonula occludens-1 (ZO-1) and claudin-14 expression in vitro and by downregulating the leaky protein claudin-2 in human intestinal epithelial cell culture monolayers. Another study conducted by the same authors [6] showed that EVs isolated from EcN and ECOR63 protected the gut epithelial barrier in human intestinal epithelial cell culture monolayers infected by enteropathogenic *E. coli* (EPEC). Among the effects observed, EcN and ECOR63 EVs prevented tight junction protein redistribution and the protein down-regulation induced by EPEC, resulting in the neutralization of the decrease in intestinal permeability [6]. In this regard, Ashrafian et al. [9] evaluated the effect of EVs isolated from the commensal bacterium *Akkermansia muciniphila* in the human intestinal epithelial cell culture model Caco-2. Remarkably, the authors observed that *A. muciniphila* EVs enhanced tight junction gene expression and down-regulated toll-like receptor (TLR) gene expression, which suggested a potential role for *A. muciniphila* EVs in intestinal barrier permeability and fortification as well as in inflammation reduction [9]. The protective role against inflammatory responses mediated by commensal and/or probiotic bacterial EVs was ratified by the studies of Hiippala et al. [59], which showed that commensal *Odoribacter splanchnicus* 57 EVs possessed immunomodulatory properties, mitigating the production of the pro-inflammatory cytokine IL-8 in enterocyte cultures treated with *E. coli*-lipopolysaccharide (LPS). The anti-inflammatory properties of *O. splanchnicus* could potentially rely on sphingolipids, such as sulfobacin B [59]. In addition, Kang et al. [117] also showed that *A. muciniphila* EVs pre-treatment of a colon epithelial cell line could hinder the upregulation of pro-inflammatory cytokines induced by pathogenic *E. coli* EVs. The role of gut microbiota and probiotic EVs on modulating intestinal inflammation and gut barrier integrity has been further confirmed by several in vivo studies. For example, Kang et al. [117] provided direct evidence that oral administration of *A. muciniphila* EVs elicited protective effects, attenuating the severity of colitis-associated phenotype in mice. These effects involved the reduction of weight loss and gut barrier disruption and the control of inflammatory response [117]. In fact, an independent study carried out by Fábrega et al. [46] also described immunomodulatory properties for orally administered probiotic EcN EVs, which protected against the development of experimental-induced colitis in mice by counteracting weight loss, inflammation, and gut barrier damage.

It should be noted that gut microbiota EVs could not only exert immunomodulatory functions through their interaction with gut barrier cells, but they could also communicate with intestinal immune system cells, thus influencing host immunity. In this regard, Bulut et al. [4] determined that EVs derived from the commensal bacterium *Pediococcus pentosaceus* triggered immuno-suppressive responses both in vitro and in vivo. Using several mouse models of acute inflammation, the authors showed that *P. pentosaceus* EVs promoted immune tolerance and inhibited inflammation through the direct interaction with bone marrow-derived macrophages and bone marrow progenitors by a TLR-2-dependent mechanism, which stimulates the generation of inflammation regulatory cells (M2-like macrophage and myeloid-derived suppressor-like cells) [4]. The protective effect of gut microbiota EVs against inflammatory disorders was also corroborated by Shen et al. [113], who demonstrated that *Bacteroides fragilis* EVs prevented colitis development in mice by inducing immune tolerance through activation of dendritic cells [113]. It was also suggested that the immunomodulatory effects of *B. fragilis* were mediated by a capsular polysaccharide packaged in EVs [113].

### Pro-inflammatory effects of gut microbiota– and probiotic-derived extracellular vesicles

Although gut microbiota and probiotic-derived EVs have shown anti-inflammatory effects, other studies have also attributed a pernicious role for some gut microbiota and probiotic EVs in triggering pro-inflammatory responses. For example, Patten et al. [101] demonstrated that EVs derived from the commensal bacterium *E. coli *C25 could promote pro-inflammatory responses in intestinal epithelial cells in vitro by promoting IL-8 secretion. In addition, Cañas et al. [17] unveiled that EVs derived from the probiotic EcN and the commensal gut microbiota bacterium ECOR12 could enhance the secretion of pro-inflammatory cytokines IL-6 and IL-8 in Caco-2 cells, which suggests a role for the EVs of these strains in the modulation of intestinal immune responses. Finally, Fábrega et al. [45] reported that EVs from EcN and ECOR12 could induce immune and defense responses in a human ex vivo model of colonic explants. The authors suggested that outer membrane LPS in EVs could be an explanatory mechanism for the modulation of immune system responses [45]. Among the mechanisms involved, EcN and ECOR12-derived EVs upregulated the chemokine MIP1α, the pro-inflammatory cytokines TNF-α, IL-6, IL-8, and IL-10, and the human beta-defensin-2 (HBD-2), an antimicrobial host defense peptide involved in intestinal barrier function [48]. They also enhanced IL-22 production, which has been involved in intestinal barrier protection and its strengthening [45, 86]. It should be highlighted that the induction of moderate inflammatory bowel responses is critical for gut barrier protection [120]. In fact, it has been suggested that gut microbiota–derived EVs with pro-inflammatory properties could be mild enough to be beneficial and contribute to gut barrier integrity and intestinal homeostasis maintenance through the induction of host immune and defense responses [17, 45]. Nevertheless, in a context of dysbiosis or host susceptibility to develop inflammatory disorders, we speculate that an imbalance in the abundance of pro-inflammatory EVs from gut microbiota, a priori to be beneficial to the host, may potentially lead to exacerbated inflammatory responses eventually harmful to the host. In support of this hypothesis, Hickey et al. [58] observed that EVs derived from the commensal *Bacteroides thetaiotaomicron* could be uptaken by intestinal macrophages and elicit inflammatory response by inducing pro-inflammatory cytokines (such as TNF-α and IL-1β) production, driving colitis development in genetically susceptible mice.

### Direct evidence of the effects of gut microbiota and probiotic-derived extracellular vesicles on obesity and diabetes pathophysiology

Remarkably, recent reports have confirmed the direct connection between modulation of intestinal immune responses and gut barrier integrity to gut microbiota–derived EVs, as well as their link to metabolic function, including improvements of metabolic diseases, such as obesity and diabetes. In this regard, Chelakkot et al. [23] reported a direct relationship between *A. muciniphila* EVs and the improvement of both gut barrier integrity and metabolic profile in high-fat diet (HFD)–induced diabetic mice. Thereby, oral administration of *A. muciniphila* EVs to HFD-fed mice decreased gut barrier permeability, reduced body weight gain, and improved glucose tolerance [23]. In addition, administration of *A. muciniphila* EVs to LPS-treated Caco-2 cells decreased intestinal permeability and improved intestinal barrier integrity through the up-regulation of occludin expression [23]. These findings led the authors to propose that *A. muciniphila* EVs could be a therapeutic tool to treat metabolic diseases such as obesity and diabetes [23]. This hypothesis was confirmed by Ashrafian et al. [8] who demonstrated an effect of *A. muciniphila* EVs on the alleviation of obesity in mice. *A. muciniphila* EVs reduced food intake, body weight, and adiposity; increased gut barrier integrity; ameliorated intestinal and adipose tissue inflammation; and improved metabolic function in HFD-induced obese mice [8]. Indeed, it could be suggested that the improvements on host metabolic health due to *A. muciniphila* [35] and other gut microbiota species might be mediated, at least partially, through dynamic EV production.

In contrast, Choi et al. [25] revealed the implication of gut microbiota–derived EVs in the worsening of diet-induced metabolic disorders, in the context of gut microbiota dysbiosis. After investigating the effect of stool EVs isolated from HFD-fed mice, the authors observed that these EVs could blunt glucose metabolism by promoting insulin resistance in both skeletal muscle and adipose tissue [25]. This study demonstrated that *Pseudomonas panacis* LPS-containing EVs were more abundant in HFD-fed mice and they mediated the detrimental effect of the HFD on glucose metabolism [25]. Remarkably, the alteration of gut microbiota EV profile was more severe than changes in gut microbe composition and, unlike gut microbes, gut microbiota EVs could penetrate into the bloodstream and arrive to insulin-sensitive tissues [25]. This result suggested that HFD-induced gut microbiota dysbiosis could be accompanied by changes in gut microbiota EV profile that could have detrimental effects on energy homeostasis, and thus contribute to the onset and progression of metabolic diseases such as obesity and diabetes. Notably, the remarkable role of commensal bacteria-derived EVs on host physiology and their contribution to the pathogenesis of dysbiosis-related diseases have been also elucidated by the studies of Yang et al. [134] in the lung commensal microbiota. In a mouse model of experimental lung fibrosis, the alteration of lung microbiota promoted IL-17B production, contributing to the pathogenesis of the disease, and such effect was mediated by EVs secreted by commensal bacteria of the genera *Bacteroides* and *Prevotella*, whose abundance was up-regulated in this pathophysiological context [134].

Some pieces of evidence dealing with the potential effect of gut microbiota and probiotic bacteria–derived EVs on host physiology (including shaping of energy metabolism, immune responses, and gut barrier function) are summarized in Table 1.

**Table 1:** Some pieces of evidence of the effect of extracellular vesicles derived from gut microbiota and probiotics bacteria on host health

Bacteria-derived EVs	Model	Mediated effect	Mechanism	Refs
*Escherichia coli* Nissle 1917 and ECOR63–derived EVs	T-84 and Caco-2 cells (colon cell lines) monolayers	Reinforcement of gut barrier (↑ TER)	(1) ↑ ZO-1 and claudin-14 (2)↓ Claudin-2	[5]
*Escherichia coli *Nissle 1917 and ECOR63–derived EVs	T-84 and Caco-2 cells monolayers infected by EPEC	Protection against EPEC-induced epithelial barrier permeability increase	Counteraction of EPEC-induced: (1) ↓ Occludin and claudin-14 gene expression (2) ZO-1 and occludin redistribution (3) F-actin cytoskeleton disorganization	[6]
*Escherichia coli* Nissle 1917 and ECOR12–derived EVs	Caco-2 cells	Modulation of innate immune responses	Activation of NF-kB thought NOD1-signaling pathway and subsequent secretion of pro-inflammatory cytokines IL-6 and IL-8	[17]
*Escherichia coli* Nissle 1917 and ECOR12–derived EVs	Human colonic explants	Intestinal barrier protection and immune and defense responses activation	↑ MIP1α, TNF- α, IL-6, IL-8, hBD-2, and IL-22 ↓ IL-12, TGF-β, and MUC1	[45]
*Escherichia coli* Nissle 1917–derived EVs	Colitis mouse model	Amelioration of experimental induced colitis progression	↓ Body weight loss ↓ Disease Activity index Counteraction of colon length decrease ↓ Intestinal damage and inflammation by counteracting altered colonic expression of pro-inflammatory cytokines (i.e., IL-1β and IL-6), and TFF-3 colonic down-regulation	[46]
*Escherichia coli* C25–derived EVs	Caco-2 and HT29-19A intestinal cells	Pro-inflammatory response	↑ IL-8 secretion	[101]
*Akkermansia muciniphila*–derived EVs	Caco-2 cells	Potential role on inflammation and intestinal barrier permeability (not determined)	↓ *Tlr2* y *tlr4* gene expression ↑ O*cldn* and *zo2* gene expression	[9]
*Akkermansia muciniphila*–derived EVs	LPS-treated Caco-2 cells	↓ Permeability	↑ Occludin expression	[23]
*Akkermansia muciniphila–derived EVs*	*Escherichia coli* EV–treated CT26 (colon epithelial cell line)	Counteraction of inflammatory response	Counteraction of pro-inflammatory cytokines production (IL-6)	[117]
*Akkermansia muciniphila*–derived EVs	Colitis mouse model	Amelioration of experimental induced colitis progression	↓ Body weight loss Counteraction of colon length decrease ↓ Epithelial barrier disruption and inflammatory cell infiltration of the colon wall ↓ Disease Activity index reduction	[117]
*Akkermansia muciniphila*–derived EVs	High-fat diet–induced diabetic mice	Improvement of gut barrier integrity Counteraction of high-fat diet–induced body weight gain Improvement of glucose tolerance	↑ Tight junction protein expression (occludin, ZO-1, and claudin-5)	[23]
*Akkermansia muciniphila*–derived EVs	High-fat diet–induced obese mice	↓ Food intake Counteraction of high-fat diet–induced body and adipose weight gain ↓ Cholesterol and plasma glucose levels ↓ Adipose inflammation ↓ Colon inflammation ↓ Gut barrier permeability	Modulation of expression of adipose genes involved in energy metabolism and fatty acid oxidation (*PPAR-α* and *PPAR-γ*) ↓ Pro-inflammatory cytokines (*TNF-α* and *IL-6*) and *TLR-4* adipose gene expression ↓ Pro-inflammatory cytokines and *TLR-4* colon gene expression ↑*TLR-2* and pro-inflammatory cytokines colon gene expression ↑ Tight junction colon gene expression (*ZO-1*, *OCLDN*, and *CLDN-1*) *↓ CLDN-2* colon gene expression	[8]
*Odoribacter splanchnicus* 57–derived EVs	HT-29 treated with *Escherichia coli*-LPS	Attenuation of *Escherichia coli* LPS-induced inflammation	Counteraction of pro-inflammatory cytokine IL-8 production	[59]
*Pediococcus pentosaceus*–derived EVs	Bone marrow–derived macrophages and bone marrow progenitors cell cultures	Inflammation inhibition by activating TLR2 signaling pathway	Induction of M2-like macrophage polarization Promotion of myeloid-derived suppressor-like cell differentiation	[4]
*Pediococcus pentosaceus*–derived EVs	Excisional wound healing model	Wound-healing rate acceleration	Inflammatory responses modulation and inflammatory suppressor cells recruitment	[4]
*Pediococcus pentosaceus–derived EVs*	Mouse models of liver fibrosis, vaccination, peritonitis, and colitis	Inflammation suppression by promoting immunosuppressive responses	Induction of M2-like macrophage polarization Promotion of myeloid-derived suppressor-like cell differentiation	[4]
*Bacteroides fragilis*–derived EVs	Colitis mouse model	Colitis development protection through inflammation suppression	↑ Anti-inflammatory cytokine and regulatory T cells production through dendritic cells TLR2-activation	[113]
*Bacterioides thetaiotaomicron*–derived EVs	Genetically susceptible- colitis mice	Development of colitis through the enhancement of inflammatory responses	Inflammatory cytokines production (IL-6 and TNF-α) by intestinal macrophages Sulfatase dependent-activation of intestinal macrophages	[58]
*Pseudomonas panacis*–derived EVs	3T3-L1 adipocytes L6 myotubes	Impairment of insulin signaling in adipocytes and myotubes Blockage of insulin uptake by myotubes	↓ Insulin signaling molecule pAKT in adipocytes and myotubes ↓ Myotubes GLUT4 translocation	[25]
*Pseudomonas panacis*–derived EVs	Mice	Diabetic phenotype induction (insulin resistance in skeletal and adipose tissues and skeletal tissue glucose intolerance)	Adipose tissue and skeletal muscle insulin signaling pathway blockage at pAKT level	[25]

### Characterization of gut microbiota and probiotic-derived extracellular vesicle cargo

The characterization of gut microbiota and probiotic-derived EV cargo has been mainly focused on the analysis of the proteome and occasionally also the lipidome and the genomic DNA [4, 14, 16, 23, 25, 45, 59, 75, 98, 113]. Of note, as previously described, the effector molecules responsible for the effect of probiotic- and gut microbiota-derived EVs have been identified [25, 45, 113]. However, the identification of bioactive molecules that mediate gut microbiota and probiotic-derived EVs is in its infancy, since most of the studies have been focused on the characterization of properties, effects, and cargo of pathogenic bacteria–derived EVs rather than commensal bacteria-derived EVs. Furthermore, as reviewed by other authors, it should be noted that bacteria-derived EVs are also enriched in numerous molecules besides proteins, like glycolipids, lipopolysaccharides, peptidoglycans, and nucleic acids (i.e., DNA and several types of RNA, such as rRNAs, tRNAs, mRNAs, and miRNAs-like molecules) [36, 130]. For this reason, a more exhaustive characterization of the different types of cargo of gut microbiota– and probiotic-derived EVs is required, since it cannot be ruled out that other bioactive molecules such as miRNAs could potentially be responsible for some of the effects of these EVs on the host.

However, there are currently limitations to characterize gut microbiota-derived EVs, which make the identification of the molecules involved in their physiological effects difficult. Several methods have been carried out to isolate and characterize bacterial EVs from in vitro cultures [55, 98]. However, the isolation and characterization of the heterogeneous profile of the gut microbiota are far more complex. As it was pointed out by other authors [95], a major obstacle is the lack of bacterial EV universal markers and their size-similarities to mammalian EVs, which complicate their separation from mammalian EVs in corporal fluids. Nevertheless, research continues to overcome these limitations, and recently Lagos et al. [75] isolated and characterized EVs from pig gut microbiota in vitro, and new methods have been developed to isolate more effectively bacterial EVs from body fluids [124]. Other authors also identified the bacterial origin of fecal EVs through the amplification of the DNA from the most common gut microbiota phyla by PCR [64]. However, exhaustive identification of the origin of fluids and fecal-derived EVs and EV-derived molecules, such as miRNAs, is still a challenge due to the complexity of the material.

#### Biological effect of miRNA contained within bacterial extracellular vesicles

It has been unveiled that bacterial EV-derived miRNAs could exert trans-kingdom gene expression regulation and influence host biological functions. For example, Choi et al. [27] identified miRNA-size small RNAs in periodontal pathogen EVs that could downregulate cytokine expression (IL-5, IL-13, and IL-15) in Jurkat T cells and predicted human immune-related target genes, suggesting a potential role in shaping host immune system responses. Han et al. [57] expanded knowledge on periodontal pathogen EVs and revealed that miRNA-like molecules of *Aggregatibacter actinomycetemcomitans* EVs could enter human macrophages and regulate gene expression, promoting TNF-α production. It was also observed that *A. actinomycetemcomitans* EVs reached the brain in mice and the exogenous RNA cargo up-regulated TNF-α expression, which led to propose a potential role in neuroinflammatory disorders [57]. Thus, it should be explored if, like pathogenic bacteria–derived EVs, gut microbiota EVs could carry small RNAs, including miRNAs, that may be involved in the crosstalk between gut microbiota and the host, as some authors have already hypothesized [11, 21, 26, 57].

In fact, the reciprocal interaction, host-gut microbiota mediated by host EV-derived miRNAs, has been also documented. Indeed, the effects of host EV-derived miRNAs on gut microbiota-gene expression regulation were described by Liu et al. [85]. This pioneering study revealed that gut epithelial cells and homeodomain-only protein homeobox (HOPX)–positive cells can secrete fecal miRNAs and influence gut microbiota gene expression, controlling gut microbiota composition and homeostasis [85]. In turn, several studies have shown that gut microbiota can affect host-miRNA expression and modulate crucial host functions, such as gut permeability, inflammation, or metabolism [34, 96, 125, 127]. A correlation between gut microbiota–induced alterations on host-miRNA profile and obesity and type 2 diabetes has been also documented in several studies [49, 83, 126]. In addition, probiotic administration has also been reported to influence host miRNA expression [105]. Thus, indirectly, it has been shown that gut microbiota and probiotics could regulate host gene expression through their influence on host-miRNA expression. However, the direct interaction on host gene expression mediated by miRNA-like molecules derived from gut microbiota and/or probiotics EVs must be proven and clarified in future experiments.

## Role of diet on the modulation of gut microbiota–derived extracellular vesicles and the potential consequences on metabolic disorder outcomes

Compelling pieces of evidence indicate that diet exerts a powerful influence on the composition, abundance, diversity, and metabolism of gut microbiota and it could promote gut microbiota changes linked to the prevention and/or treatment as well as the development and/or progression of metabolic disorders [72, 79]. In fact, it has been observed that dietary patterns influence gut microbiota populations differentially, favoring or inhibiting certain bacterial species [72, 104]. Therefore, promotion of a specific gut microbiota composition profile has been established for a wide range of diets, such as vegan/vegetarian diet, gluten-free diet, Western diet, or Mediterranean diet, which have an impact on metabolism and gut barrier and immune functions [2, 51, 110, 135]. A more thorough description of this issue has been summarized in other reviews [72, 104]. For instance, the Western diet may promote obesity development by switching gut microbiota profile towards a decrease of beneficial/protective bacteria, reducing gut microbiota diversity, and enhancing local (gut) inflammation [2, 7, 41]. By contrast, a vegetarian diet has been associated with obesity prevention in several studies, for example, by increasing the abundance of commensal beneficial bacteria (*Bacteroides*, *Prevotella*, *Clostridium *sp.) and/or decreasing diversity and inflammation-related bacteria (*Enterobacteriaceae*) [68, 89]. Also, strong adherence to the Mediterranean diet has been reported to increase the population of some beneficial bacteria, shifting microbiota status towards a healthier pattern [107]. Therefore, gut microbiota shaping through dietary interventions could be an attractive, effective, and non-invasive strategy to prevent and/or treat obesity and diabetes.

However, given the growing evidence supporting an important role for gut microbiota-derived EVs in gut microbiota-host communication, and their influence in physiological function whose disturbance has been associated with metabolic disorders (i.e., inflammation, gut permeability, or metabolism) (see the “Role of gut microbiota and probiotic-derived extracellular vesicles on inflammation, obesity, and diabetes” section), it would be worthy to explore the following hypothesis:

1. The impact of diet on gut microbiota–derived EV profile.
2. If the potential modulation of gut microbiota–derived EV profile through diet could have an impact on host physiology and contribute to the development (or treatment) of metabolic disorders.

Some of these issues are just starting to be explored and an intriguing role for diet in shaping gut microbiota-derived EV characteristics, such as their production, cargo, and composition, is emerging. Remarkably, Tan et al. [118] presented evidence supporting the influence of diet on gut microbiota EV production. These authors showed that the administration of a high-protein diet (HPD) enhanced EV production in non-dysbiotic mice, activating the gut epithelial TLR4-signaling pathway [118]. Activation of the TLR4 pathway, in turn, increased immune regulators of IgA production (in particular CCL28) that modulated host-gene expression towards an enhancement of IgA production [118]. Notably, the authors attributed the gut microbiota–derived EV production enhancement to the HPD-induced upregulation of succinate [118]. Of note, IgA controls bacteria-host interactions and is implicated in the maintenance of gut homeostasis by, for instance, restricting spatially pathogens and pathogenic microbiota, affecting bacterial viability, and modulating pathogenicity [84, 99]. Indeed, Luck et al. [87] observed that HFD decreased IgA production in mice and attributed a functional role for IgA on glucose homeostasis maintenance, gut and adipose tissue inflammation, gut permeability, microbiota encroachment, and microbiota composition, which suggested an implication for IgA downregulation in HFD-induced metabolic diseases. In accordance, Tan et al. [118] also determined that HFD did not induce upregulation of succinate production and that gut microbiota-derived EVs from HFD-fed mice inhibited immune regulators of IgA production, in particular the cytokine APRIL (A Proliferation-Inducing Ligand), dramatically decreasing IgA levels. In this line, some studies have revealed that succinic acid or succinate treatments could alleviate obesity in mice [62, 128] and, as reviewed by other authors, succinate production by gut microbiota might have beneficial effects on metabolic disorders [47].

Furthermore, Lagos et al. [75] characterized the profile of EVs derived from porcine stool samples, determining that the secretion of *Clostridiales*, *Bacilli*, and *Enterobacteriales *EVs increased in response to the administration of the carbohydrate β-mannan. Indeed, β-mannan administration not only stimulated gut microbiota EV production but also modified their protein cargo [75]. However, such EVs did not carry polysaccharide- or mannan-degrading enzymes, so the relationship between β-mannan and EV production remains undermined [75]. Deep research regarding the identification of specific bacterial species whose EV production increased after β-mannan treatment, could help to elucidate the effect of β-mannan-gut microbiota EV modulation on host health.

The impact of diet on the progression of metabolic disorders through shaping gut microbiota–derived EVs characteristic has been partially revealed by Choi et al. [25]. These authors reported that administration of a HFD to mice altered the amount, size, and global content of gut microbiota EVs [25]. Thus, the characterization of EVs of HFD-fed mice stools showed a size decrease of EVs and changes in the global protein content profile, marked by an increase in LPS, due to the enhancement of EVs derived from LPS-expressing Proteobacteria [25]. In this context, other studies have observed that LPS (eventually present in bacteria-derived EVs) could increase in response to a HFD and have identified LPS as a key triggering factor in diabetes and obesity onset [19]. LPS might increase metabolic endotoxemia, which in turn could disrupt energy homeostasis, promote inflammation, induce insulin resistance and diabetes, and enhance body weight gain in mice [19]. Thus, the enhancement of LPS-containing gut microbiota EVs could potentially be an important underlying mechanism that could orchestrate the promotion of metabolic endotoxemia and metabolic disorders induced by HFD. In this sense, it has been reported that blood levels of LPS-containing EVs are higher in patients with intestinal barrier dysfunction; and these EVs strongly stimulated secretion of proinflammatory cytokines [123]. On the other hand, gut barrier permeability may be compromised in patients with obesity and type 2 diabetes and dietary lipids could aggravate permeability disturbance [52]. Therefore, it would be necessary to confirm in humans (1) if gut barrier disruption could enhance the translocation of LPS-positive bacterial EVs, (2) if a Western diet could enhance the production of these EVs, and (3) further clarify the implication of EVs from LPS-positive bacteria in human metabolic disorders, as it was previously described in mice.

Interestingly, Müller et al. [92] also showed that probiotic-Lactobacilli EV properties could be shaped by their living microenvironment. It was found that culture conditions, agitation, and pH 5, respectively, modified *Lactobacillus casei* and *Lactobacillus plantarum* EV protein content and strengthened their anti-inflammatory properties, leading to the enhancement of IL-10 and TNF-α production after macrophage treatment in vitro [92]. In this context, accumulating evidence has demonstrated that EVs derived from bacteria commonly used as probiotics possess anti-inflammatory properties and could be potentially used to prevent or treat inflammatory disorders [28, 38, 71]. For instance, it has been described that *Lactobacillus paracasei* EVs counteracted LPS-induced human colorectal cell inflammation in vitro and attenuated inflammation responses and ameliorated colitis in mice, suggesting an important role in gut homeostasis maintenance [28]. In addition, oral administration of EVs derived from *A. muciniphila* displayed immunomodulatory properties and ameliorated HFD-induced obesity in mice [8]. On the other hand, several dietary ingredients beneficially affect the growth, survival, and/or activity of probiotic bacteria [42, 111]. This finding provides a new avenue to analyze if specific food ingredients, in a similar manner as culture conditions, could enhance probiotic EV immunomodulatory properties and if these EVs with strengthened anti-inflammatory effects could be used to effectively treat metabolic diseases, in which inflammation is a hallmark [61].

### Effect of diet on gut microbiota through food-derived extracellular vesicles

It is worthy of note that a substantial amount of studies have shown that dietary EVs can directly interact with mammalian cells, such as tumor cells, epithelial cells, immune system cells, or hepatocytes, and they could elicit biological functions that could have an impact on host health, even emerging as potential therapeutic targets for the treatment of a wide range of diseases, such as inflammatory disorders, cancer or steatosis [102, 106]. In addition, dietary EVs could be also prominent players in the interaction between diet and gut microbiota, which suggests that dietary EVs might be implicated in the extensively described effect of diet on host physiology. In support of this notion, it has been reported that dietary bovine milk exosomes (a particular type of EV) could modulate gut microbiota composition, which was associated with the increase of short-chain fatty acids (SCFAs) levels [121]. In addition, the authors identified the consequences of exosome-induced gut microbiota modulation in mice, suggesting an effect on the maintenance of intestinal barrier function and intestinal immune system regulation [121]. Notably, results provided by other studies have revealed that gut microbiota SCFAs could be useful in the improvement of obesity and diabetes [18, 82], but a direct connection between the modulation of SCFA production by dietary EVs and metabolic counteraction remains to be established. However, the direct effect of bovine milk exosomes on human health is unclear, since it has been also suggested that they could contribute to the pathogenesis of obesity and type 2 diabetes [90]. This might be due to the fact that several of the most abundant miRNAs of bovine milk–derived exosomes could disrupt energy homeostasis, influencing processes such as adipogenesis, insulin secretion, or glucose transporters [90]. In addition, it has been shown that dietary plant–derived EVs could also shape gut microbiota and accumulated evidence suggests that exogenous miRNAs (xenomiRs from foods) within EVs could influence human health, particularly involving gut microbiota and intestinal barrier function [40]. Recently, Teng et al. [119] observed that ginger exosome-like nanoparticles, through miRNAs, could regulate *Lactobacillus rhamnosus* (LGG) gene expression, influencing the intestinal immune system and improving gut barrier integrity, ameliorating colitis in mice [119]. It has been reported that LGG administration has anti-obesity and anti-diabetic properties, being able to alter gut microbiota composition, decrease weight gain, inhibit leptin resistance, enhance glucose tolerance and insulin sensitivity, and reduce adiposity [24, 63, 69, 100]. Thus, manipulation of gut commensal LGG through dietary ginger exosome–like nanoparticles containing miRNAs may be a promising strategy to treat metabolic syndrome. Remarkably, Berger et al. [13] provided direct evidence of the functional consequences of plant-derived EVs in obesity and predicted potential EV-encapsulated miRNAs that might be involved in the observed effects, but it has not been explored whether these EVs could also target gut microbiota [13].

## Conclusions and future directions

The use of probiotics to treat metabolic disorders in order to enhance weight loss and to improve host homeostasis has been an interesting strategy to achieve optimal metabolic health reviewed by several authors [76, 109]. Alternatively, there is compelling evidence regarding the emergence of probiotic- and gut microbiota–derived EVs as a potentially safe and effective alternative as compared to probiotics, as discussed in recent reviews [91]. In line with these findings, in 2013, the European Medicines Agency (EMA) approved the bacterial-derived 4CMenB vaccine, which is a bacteria extracellular vesicle–based vaccine against meningococcal infection for children [15]. Indeed, several authors hypothesized that, considering the efficacy and safety of this vaccine, likewise, bacterium-derived EVs could be potentially used as drugs and could be administered to modulate host physiology and to treat inflammatory diseases [4]. The potential of a new formulation based on probiotic EVs, called probiomimetics, as a therapeutic tool to treat inflammatory diseases, has been already shown [74]. The probiomimetics are engineered therapeutic agents that consist of microvesicles with surface-attached probiotic EVs [74]. Nevertheless, further research is needed to clarify whether the observed effects of probiotics on improving diet-induced gut microbiota dysbiosis [73, 116] could be mediated by their EVs, and whether the administration of gut microbiota and probiotic EVs could be used as an emerging tool for the prevention and management of metabolic diseases, since the available evidence in this field is limited. In addition, exhaustive characterization of the cargo of probiotic- and gut microbiota–derived EVs is still required to identify all the bioactive molecules (i.e., miRNAs) responsible for their biological effects.

On the other hand, some questions that are poorly understood regarding the effects of diet on shaping gut microbiota EVs should be elucidated, which include the following:

1. To determine to which extent the contribution of diet-induced microbiota dysbiosis to obesity and diabetes pathogenesis is mediated by the alteration of the microbiota EV profile.
2. To identify dietary patterns that could potentially lead to the establishment of a healthy (i.e., Mediterranean or vegetarian diets) or detrimental (i.e., Western or HFD) gut EV profile.
3. To favorably modulate the gut microbiota EV profile through the administration of certain bacterial EVs, probiomimetics, bioactive compounds, or foods that could represent new approaches to tackle metabolic diseases.

**Fig. 1:**
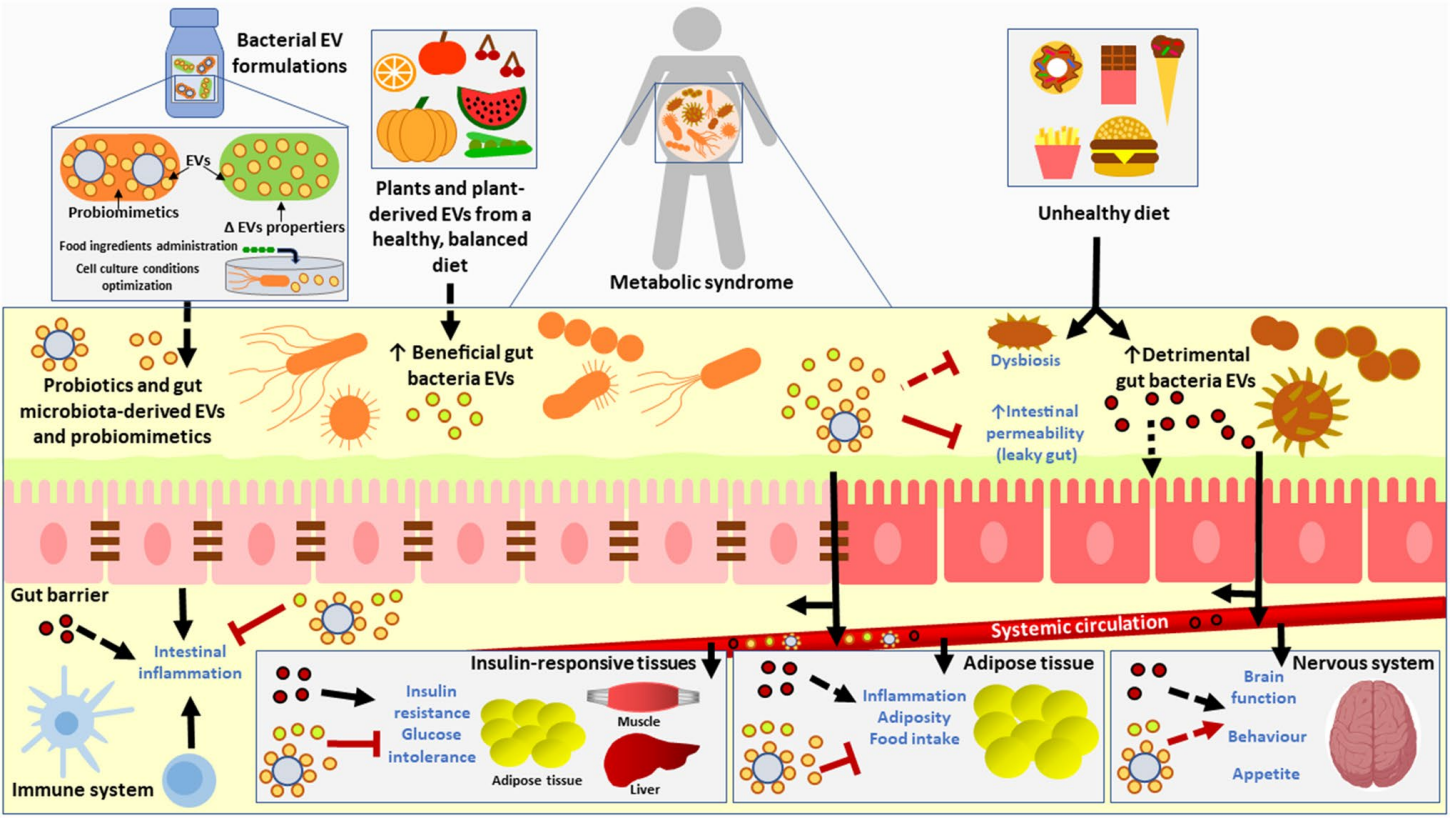
Schematic model of the hypothetical impact of dietary patterns on gut microbiota extracellular vesicle profile, and the effect of probiotic- and gut microbiota–derived extracellular vesicles on metabolic syndrome onset and/or progression. A mechanism by which unhealthy dietary patterns could contribute to obesity and/or diabetes progression could be the promotion of gut microbiota dysbiosis and the enhancement of detrimental gut microbiota-derived extracellular vesicles amount. In turn, these extracellular vesicles could potentially impair gut barrier permeability and intestinal inflammation, and could be distributed to peripheral organs through systemic circulation, impairing metabolism and promoting inflammation, and subsequently aggravating the severity of metabolic disorders. Enhancement of beneficial gut microbiota extracellular vesicles through plants and plant-derived extracellular vesicles from healthy dietary patterns and/or the administration of formulations based of probiotic- and gut microbiota–derived extracellular vesicles could potentially be a strategy to treat metabolic disorders by counteracting dysbiosis, gut permeability increase, inflammation, metabolic homeostasis disturbances, and nervous-system derangements. Formulations based on bacterial extracellular vesicles could be optimized in order to increase their properties and effects by designing efficient therapeutic agents (i.e., probiomimetics, which are bacterial derived extracellular vesicles coupled to microvesicles), establishing optimal cell culture conditions or administrating certain food-ingredients. All the hypotheses presented in this model must be further confirmed; especially the ones marked with dashed arrows should be proven. Brain template from BioRender (https://biorender.com)

Improving the knowledge of the mechanisms underlying the crosstalk between diet, gut microbiota, and the host would allow the identification of new therapeutic targets for obesity and diabetes prevention and management. As presented in Fig. 1, gut microbiota–, probiotic-, and food-derived EVs could be a key factor of this interaction. Administration of probiotic- and commensal bacteria-derived EVs and/or manipulation of gut microbiota-derived EVs through dietary interventions should be explored as potential strategies to prevent and treat metabolic disorders.

## Data Availability

Not applicable.
